# Clotting and Proteolytic Activity of Freeze-Dried Crude Extracts Obtained from Wild Thistles *Cynara humilis* L. and *Onopordum platylepis* Murb.

**DOI:** 10.3390/foods12122325

**Published:** 2023-06-09

**Authors:** Cindy Bande-De León, Laura Buendía-Moreno, Adela Abellán, Pamela Manzi, Bouthaina Al Mohandes Dridi, Ismahen Essaidi, Lucia Aquilanti, Luis Tejada

**Affiliations:** 1 Department of Human Nutrition and Food Technology, Universidad Católica de Murcia-UCAM, Campus de los Jerónimos, 30107 Murcia, Spain; aabellan@ucam.edu (A.A.); ltejada@ucam.edu (L.T.); 2 Department of Food Science and Technology, Veterinary Faculty, University of Murcia, 30071 Murcia, Spain; 3 CREA, Centro di Ricerca Alimenti e Nutrizione, Via Ardeatina 546, 00178 Rome, Italy; pamela.manzi@crea.gov.it; 4 Research Laboratory of Agrobiodiversity and Ecotoxicology LR21AGR02, High Agronomic Institute of Chott-Mariem, University of Sousse, Sousse 4042, Tunisia; bouthaina2@yahoo.com (B.A.M.D.); saidi.ismahen@gmail.com (I.E.); 5 Departament of Agricultural, Food and Environmental Sciences, Università Politecnica delle Marche, Via Brecce Bianche, 60131 Ancona, Italy; l.aquilanti@staff.univpm.it

**Keywords:** milk clotting activity, vegetable coagulant, proteolytic activity

## Abstract

The rising interest in finding alternatives to animal rennet in cheese production has led to studying the technological feasibility of using and exploiting new species of herbaceous plants. In this research work, and for the first time, freeze-dried extracts from *Cynara humilis* L. (CH) and *Onopordum platylepis* Murb. (OP) were studied for mineral and protein content, and their clotting and proteolytic activity were compared to those of *Cynara cardunculus* L. (CC). The effect of extract concentration (5–40 mg extract/mL), temperature (20–85 °C), pH (5–8), and CaCl_2_ concentration (5–70 mM) on the milk clotting activity (MCA) of CC, CH and OP extracts was evaluated. The MCA values were significantly higher in CC at the same extract concentration. The extract that showed the most significant increase in clotting activity due to increased temperature was OP, with maximum activity at 70 °C. The pH value for maximum milk clotting was 5.0 for both CC and CH, whereas, in the case of OP, the pH value was 5.5. CaCl_2_ enhanced the clotting capacity of the extracts, particularly for OP and CH. Furthermore, proteolytic activity (PA) and the hydrolysis rate increased with increasing time and enzyme concentration, with CC being the extract that achieved the highest caseinolytic activity.

## 1. Introduction

The enzymatic clotting of milk is an essential step in most cheesemaking processes. One of the main proteases responsible for this coagulation is chymosin, which is present in ruminant rennet and has been used for centuries by the dairy industry to manufacture different types of cheese [1].

Most of the commercial animal rennet used in dairies comes from recombinant sources, while only 20–30% of it is of natural origin [2]. The worldwide increase in cheese production has led to a decrease in the supply of animal rennet, thus increasing the demand for new coagulant substitutes, such as plant-derived proteases [1].

These proteases are obtained via maceration in water of different plant sections or organs (seeds, flowers, fruits, rhizomes, etc.) and have a high potential for use as milk coagulants to manufacture cheese, replacing animal rennet [3,4,5,6,7].

Numerous studies have been carried out on these plant enzymes obtained from aqueous extracts, such as those derived from papaya (papain), pineapple (bromelain) [8,9], and other plants, whose enzymes are characterised by a proteolytic to clotting activity ratio that is not sufficiently high or proteolytic activity that is excessively high for the production of commercial cheeses [10]. In addition, these vegetable coagulants have certain limitations, mainly related to the texture and sensory quality of the produced cheeses [3].

However, cheeses made with plant proteases from the genus *Cynara* L. have a smooth, creamy texture and exquisite flavour, thus explaining why these perennial herbaceous plants have been used since ancient times to produce traditional cheeses [11].

The genus *Cynara* L. is native to the Mediterranean flora and belongs to the family Asteraceae; it encompasses eight species and four subspecies, including *Cynara cardunculus* L. (CC) and *Cynara humilis* L. (CH). Some of these *Cynara* species have traditionally been used as milk coagulants in cheesemaking due to their high concentration of proteolytic enzymes responsible for clotting, such as cyprosins or cardosins [12]. These enzymes, present in the characteristic violet flowers that are dried and macerated for use, have proven to be successful substitutes for animal rennet [6]. CC has been used since ancient times for the manufacture of goat and sheep cheeses in several rural areas of Spain and Portugal [13]; some examples are Los Pedroches (Córdoba), Torta del Casar (Cáceres), La Flor de Guía (Gran Canaria), Serra da Estrela (Portugal), and Serpa (Portugal), among others [14].

Various studies have shown that the use of aqueous extracts obtained via the maceration of thistle flowers increases the initial microbial count of the milk and, consequently, of the curd [15,16]. Subsequent studies have shown that freeze drying aqueous extracts does not alter the initial microbial count after addition and have recommended its use as it is soluble in water and milk, is free of viable micro-organisms, has a stable shelf life without the need for preservatives, and has a coagulating activity that does not decrease after one year of storage [7,17,18,19].

The available studies evaluating the milk clotting activity (MCA) of freeze-dried thistle extracts and their use in cheese production have only been assessed in CC [7,17,18,19,20,21,22].

The genus *Onopordum* L. belongs to the family Asteraceae, and some of its species are widely distributed in Europe, Northern Africa, the Canary Islands, and the Caucasus, as well as Southwest and Central Asia. Thistles within this genus are native mainly to the Mediterranean biogeographical region and have been studied for their potential as antimicrobial, haemostatic, and hypotensive agents [23,24].

Very few studies describe the use of *Onopordum* spp. in cheese production. Very recently, Mozzon et al. (2020) [25] and Foligni et al. (2022) [26] studied the milk clotting and the caseinolytic activity of a freeze-dried extract from *Onopordum tauricum* Willd. in milk of different origins. To the author’s knowledge, no study has evaluated the milk clotting activity (MCA) and proteolytic activity (PA) of the species *Onopordum platylepis* Murb.

Optimal conditions for the milk clotting of aqueous extracts from flowers of *C. cardunculus* L., *C. humilis* L. and *C. scolymus* L. have already been described [27,28,29,30], as well as has the proteolytic activity of cardosins A and B on goat casein [31], the proteolytic activity of *C. humilis* L. on ovine Na-caseinate [32] and of *C. scolymus* L. flower extract on bovine casein [33]. Available studies on CH describe the effect of pH and temperature on the rheological properties of gels curdled with CH [34,35].

However, as far as the authors know, no research studies are available on freeze-dried CH and OP performance as agents with clotting and caseinolytic activity.

The main objective of this research work was to characterize the MCA (the effect of extract concentration, temperature, pH, and calcium chloride (CaCl_2_) concentration) and PA of freeze-dried extracts obtained from CH and OP compared to those obtained from CC. In order to characterize the freeze-dried extracts, the mineral and the protein content of the extracts obtained from CH and OP were also reported.

## 2. Materials and Methods

### 2.1. Freeze-Dried Enzymatic Extract Preparation

CC (from Cáceres, Extremadura, Spain), CH (from Cáceres, Extremadura, Spain), and OP (from Sousse, Tunisia, Africa) were the thistle species used for the preparation of the freeze-dried crude extracts. These were later freeze-dried following the procedure described by Tejada and Fernández-Salguero (2003) [22]. The plant material, styles, and stigmas were macerated in distilled water for 24 h at 25 °C in a 1:10 (*w*/*v*) ratio. The aqueous extract obtained was sieved, and the filtrate was centrifuged at 3000× *g* for 5 min. The supernatant obtained was filtered with Whatman No. 1 paper. The filtrate obtained was frozen at −32 °C for 24 h and then lyophilised (Alpha 1-2LD plus, Christ, Osterode am Harz, Germany) at a working pressure between 4 and 13 Pa. The lyophilised powder was hermetically packed and frozen at −20 °C until its use.

### 2.2. Characterisation of Thistle Extracts

#### 2.2.1. Total Protein Content

The total protein of CC, CH, and OP was determined according to the Bradford method [36] using the Sigma (Sigma-Aldrich, Milan, Italy) ready-to-use reagent. A set of bovine serum albumin (Merck, KGa, Darmstadt, Germany) solutions (0.1–1 mg/mL) was used for calibration. Absorbance readings at 595 nm were carried out using a UV-1800 Shimadzu (Kyoto, Japan) spectrophotometer. The protein content of the three enzymatic extracts studied was determined in triplicate.

#### 2.2.2. Mineral Content

Ca, P, Na, K, Mg, Zn and Mn contents of the freeze-dried crude extracts from CH and OP were determined according to the AOAC (2002) [37] method. Briefly, 0.3 g of each sample was ashed. After mineralisation, samples were solubilised in 1 mL of HNO_3_ 65% and then adjusted to a final volume of 50 mL of 1% HNO_3_ (*v*/*v*) with 0.1% (*w*/*v*) CsCl to avoid sodium and potassium ionisation and with 0.1% LaCL_3_ (*w*/*v*) for Ca and Mg detection. Ca, Na, K, Mg, Zn and Mn were detected using Atomic Absorption Spectrometer A. Analyst 300 (Perkin Elmer, Norwalk, CT, USA), while phosphorus content was measured at 400 nm using a UV-1800 spectrophotometer (Shimadzu Corporation, Tokyo, Japan). NIST, SMR1570a and SMR1846 (only for Mg) were used as standard reference materials. 

#### 2.2.3. Milk Clotting Activity Assay

The milk clotting activity (MCA) of the extracts was assessed using the Berridge test according to the International Dairy Federation (IDF) [38] based on the visual evaluation of the first clotting flakes’ appearance. For the clotting activity determination, 10 mL of reconstituted skimmed milk powder (0.12 kg/L) was transferred into a clean and dry test tube. A calcium chloride solution was added at the concentration established in each test (Sigma-Aldrich, Milan, Italy). The assay tube was allowed to equilibrate for 5 min at the desired temperature in an M20 thermostatic water bath (Lauda-Konigshofen, Germany) before adding the enzymatic extract (CC, CH, and OP). After reaching the temperature, 0.1 mL of the enzymatic extracts were added. The time from the addition of the enzyme to the first appearance of solid material was measured in seconds, as clotting. One Soxhlet unit (SU/mL) of clotting activity was defined as the volume of milk that can be clotted by one volume unit of the enzymatic extract in 40 min under defined temperature, pH, and CaCl_2_ test conditions [25] and was calculated with the following equation:

(1) $$MCA\left(SU/mL\right)=\left(2400\times M\right)÷\left(T\times V\right)$$

where M is the volume of milk (mL); T is the clotting time in seconds; and V is the volume of the enzyme (mL).

The effect of four independent variables was studied (extract concentration (5–40 mg/mL), temperature (20–85 °C), pH (5–8), and CaCl_2_ concentration (5–70 mM)) on the milk clotting activity. The clotting activity of the three enzymatic extracts studied was determined in triplicate.

To measure the effect of extract concentration (5, 10, 20, 30 and 40 mg/mL), the conditions of temperature (32 °C), pH (6.2), and CaCl_2_ concentration (10 mM) were set.

The effect of temperature (20, 25, 30, 35, 40, 45, 50, 60, 70, 80, and 85 °C), pH (5, 5.5, 6, 6.5, 7, 7.5 and 8) and CaCl_2_ concentration (5, 10, 15, 20, 30, 50, 50, 60 and 70 mM) variables were measured at two extract concentrations (20 and 40 mg/mL).

#### 2.2.4. Proteolytic Activity

The proteolytic activity of the enzymatic extracts was determined following the method employed by Silva and Malcata (2005) [39]. The substrate used was bovine milk casein, free of carbohydrates and fatty acids (Calbiochem, Darmstadt, Germany) at 1% (*w*/*v*) in a 10 mM citrate buffer (pH 6.2) (Sigma-Aldrich, St. Louis, MO, USA) with 0.03% (*w*/*v*) sodium azide (Fisher Scientific, Madrid, Spain) to avoid microbial growth, and was incubated in a bath at 32 °C. Hydrolysis was started by adding 0.12 mL of the reconstituted extract at different concentrations (5, 10, 20, 30 and 40 mg of freeze-dried extract/mL) to 3 mL of the casein solution. Subsequently, 0.5 mL aliquots were sampled at different times (5, 10, 20, 30, 40, 50 and 60 min) and put in Eppendorf tubes. The proteolytic activity was quantified via an evaluation of the peptides soluble in aqueous 5% (*w*/*v*) trichloroacetic acid (TCA) (Sigma-Aldrich, St. Louis, Missouri, USA). For this, 1 mL of 5% TCA (*w*/*v*) was added to each tube, incubated for 10 min at 25 °C, and centrifuged at 12,000× *g* for 10 min, while the absorbance of the supernatant was measured at 280 nm in a quartz cuvette. A proteolytic unit (U) was defined as the amount of enzymatic extract that produced a 0.001 unit increase in absorbance at 280 nm per minute under the stated test conditions. All determinations were made in triplicate.

### 2.3. Statistical Analysis

All experiments were conducted in triplicate, and the results were expressed with the mean and standard error. The statistical analysis of different parameters was computed using the SPSS version 21.0 software package (IBM Corporation, Armonk, NY, USA). In order to assess differences between the species, a one-way analysis of variance (ANOVA) was applied to mineral composition. Regarding the MCA analysis, a two-way ANOVA was performed to study the influence of temperature, pH, and CaCl_2_ concentration. For PA, a two-way ANOVA was applied to analyse the effect of the species and reaction time. Tukey’s HSD test (*p* < 0.05) was performed to determine significant differences between the treatment groups. Differences were considered statistically significant when *p*-values were equal to or below 0.05. Relationships among the studied factors are presented using appropriate curves and tables.

## 3. Results and Discussion

### 3.1. Total Protein Content

The protein content of the reconstituted freeze-dried extracts was different (*p* < 0.05) between species CC, CH, and OP, corresponding to 0.1018 ± 0.0065 (mean ± standard error), 0.1121 ± 0.0102 (mean ± standard error) and 0.0764 ± 0.0011 (mean ± standard error) mg protein/mg extract, respectively.

### 3.2. Mineral Content

In Table 1, the mineral contents of the freeze-dried extracts from CH and OP are shown; in more detail, CH showed a higher content of calcium, potassium, magnesium, and zinc (*p* < 0.05) than OP did, while phosphorous and manganese contents were higher in OP. No differences were seen between these two species for sodium content.

To the authors’ knowledge, very scarce data are currently available in the literature about the mineral composition of thistle extracts and, above all, about the mineral composition of CH and OP. A recent study [26] analysed the mineral composition of a freeze-dried extract prepared from *Onopordum tauricum*; the results of this investigation are consistent with those herein reported for OP, especially for P, K, and Zn content.

Some currently available data refer to *C. cardunculus* L. subsp. *scolymus* (L) and *C. cardunculus* L. var. *altilis* (DC) leaves, which have been recognised as a good source of K and Ca; nevertheless, among the micronutrients, mainly Fe and Zn, the mineral composition of thistle leaves is strictly affected by the concentration of nutrient solutions used for the treatment of thistles during their cultivation [40].

In a further investigation, the mineral content of CC flowers and seeds was also reported [41]. These vegetable parts contain considerable amounts of K, Ca, and Mg, while they are poor in Na [42]. According to Hajji Nabih et al. (2021) [43], the main micro-elements in stems of CC were Na, K, Ca, Mg, B, and P, along with other trace elements (including Zn and Mn).

### 3.3. Milk Clotting Activity Assay

The milk clotting time measured for each variable studied at the different extract concentrations is given in the Supplementary Materials (Supplementary Tables S1–S4).

#### 3.3.1. Effect of Thistle Species and Extract Concentration

**Figure 1:**
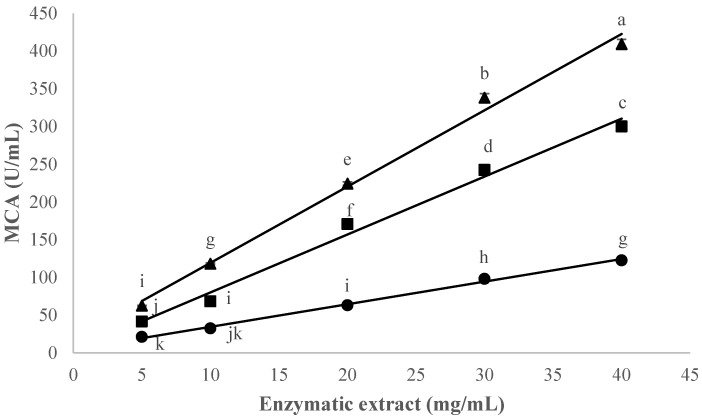
Effect of the enzymatic extract concentration and the species, expressed in mg extract/mL, on the clotting activity (MCA), expressed in SU/mL, of standard bovine skimmed milk at pH 6.2 and 10 mM CaCl_2_, using CC, (▲) CH (■) and OP (●). Data are the mean of three independent experiments (*n* = 3). Error bars correspond to standard deviations. Items with different letters (a–k) are significantly different (HSD test, *p* < 0.05).

The effect of the concentration (5–40 mg/mL) of CC, CH, and OP extracts on the clotting activity in milk at 32 °C, pH 6.2 and 10 mM CaCl_2_ is shown in Figure 1.

The milk clotting activity depends on the concentration of the enzyme. In this study, the MCA value increased with increasing extract concentration (*p* < 0.001); the concentration at which the maximum MCA was reached in all species was 40 mg/mL, and the highest MCA value (409.28 SU/mL) was seen for CC at an extract concentration of 40 mg/mL. The maximum values obtained for CH and OP were 170.64 and 63.16 (SU/mL), respectively.

The correlation between enzyme concentration and clotting time is well-known and has been studied by many authors [30,31,39,44] whose results clearly showed a decrease in clotting time as the concentration of proteases increased and are consistent with our results.

The great MCA performance of the CC species may be due to its high caseinolytic capacity and its content of chymosin-like proteases (Cardosin A and B) acting on κ-casein, more specifically on Phenylalanine_105_–Methionine_106_ bonds [45,46].

To the authors’ knowledge, no data are currently available on the performance of *Onopordum platylepis* for MCA performance. Nevertheless, studies evaluating the milk clotting properties of other subspecies of the genus *Onopordum* [25,47] also found maximum MCA values at higher extract concentrations in bovine milk.

#### 3.3.2. Effect of Thistle Species and Temperature

The effect of temperature on the clotting activity (*p* < 0.001) of the three extracts of plant origin (CC, CH, and OP) on milk at pH 6.2 and a CaCl_2_ concentration of 10 mM was evaluated at temperatures between 20 and 85 °C and different extract concentrations (20 and 40 mg/mL).

**Figure 2:**
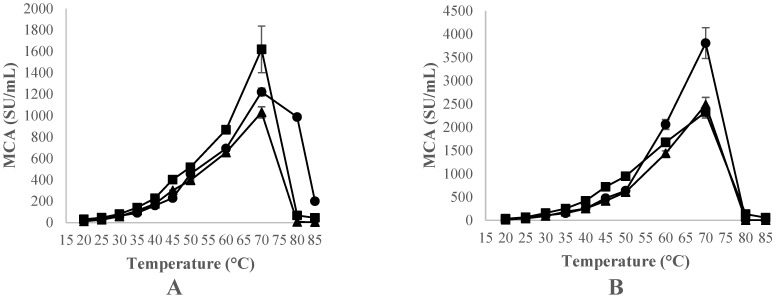
Effect of temperature, expressed in °C, on the clotting activity (MCA), expressed in SU/mL, of standard bovine skimmed milk at pH 6.2 with10 mM CaCl_2_, using CC, (▲) CH (■) and OP (●), at the concentrations of 20 mg extract/mL (**A**) and 40 mg extract/mL (**B**). Data are the means of three independent experiments (*n* = 3). Error bars correspond to standard deviations.

The clotting activity of these extracts increased with temperature, with higher coagulation developing at 70 °C in all cases. Furthermore, the milk clotting activity was influenced by the concentration of extract used, as the 20 mg/mL concentration showed lower MCA values than the 40 mg/mL extract concentration did (Figure 2).

Comparing between species, at 70 °C and at an extract concentration of 20 mg/mL, the CH extract showed significantly higher MCA (*p* < 0.05) than the other species herein assayed did. Nevertheless, the clotting capacity of the OP extract concentration was much more highly favoured by an increase in temperature than that of the other thistle species. Therefore, at the concentration of 20 mg/mL, OP showed a MCA 14.80 and 4.5 times higher than that of CH (*p* < 0.05) at temperatures of 80 and 85 °C, respectively. Moreover, the MCA for OP at 40 mg/mL was 1.23 and 1.64 times higher than that of CH at 60 and 70 °C (*p* < 0.05).

At temperatures above 70 °C, the milk clotting activity of the extracts was found to decrease in all the species herein assayed, indicating the denaturation of the enzymes.

The milk coagulating agents of plant origin assayed in this research consist of clearly thermophilic enzymes whose clotting activity increased with temperature to relatively high values. Several studies [44,48,49] reported that the optimum activity of an aqueous crude extract obtained from CC was between 40 and 60 °C; the same authors reported a decrease in the activity at temperatures over 70 °C, thus agreeing with our results. Furthermore, Ref. [50] confirmed the thermal stability of the aqueous extracts obtained from CH flowers. More recently, Mozzon et al. (2020) [25] investigated the clotting properties of a freeze-dried extract from *O. tauricum* L., observing that its optimum coagulation temperature was 55 °C, the highest in the range tested (35–55 °C), thus leading to the conclusion that temperature positively affects the MCA of the freeze-dried extract.

#### 3.3.3. Effect of Thistle Species and pH

**Figure 3:**
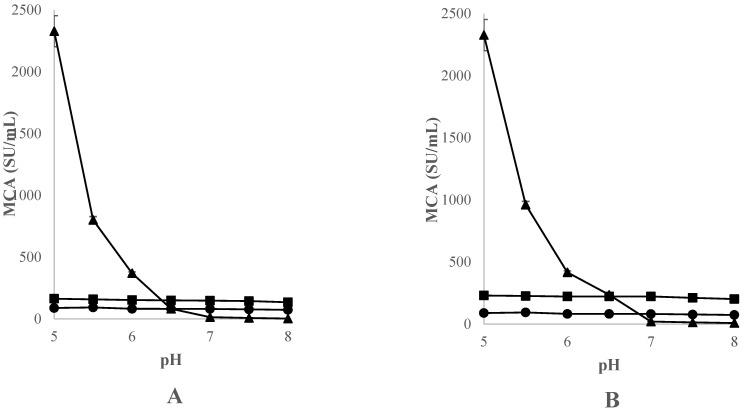
Effect of pH on the clotting activity (MCA), expressed in SU/mL, of standard bovine skimmed milk at 32 °C with 10 mM of CaCl_2_, using CC, (▲) CH (■) and OP (●), at the concentrations of 20 mg extract/mL (**A**) and 40 mg extract/mL (**B**). Data are mean of three independent experiments (*n* = 3). Error bars correspond to standard deviations.

Figure 3 shows the influence of pH on the clotting activity of CC, CH, and OP freeze-dried crude extracts, at concentrations of 20 and 40 mg extract/mL, on milk at 32 °C with 10 mM CaCL_2_.

The clotting activity of these extracts was observed to respond to a wide range of pH values (5.0–8.0), with the maximum MCA value reaching between 5.0 and 5.5. This evidence was expected since the aspartic proteinases from the Cardueae tribe have been shown to have higher milk clotting and caseinolytic activity in acidic pH ranges [39,47]. More specifically, CC and CH presented a maximum MCA at an extract concentration of 40 mg/mL and a pH of 5.0, while OP presented this at a pH of 5.5. As the pH of the milk was increased, the clotting activity of all extracts was observed to decrease drastically at both extract concentrations studied. The extract concentration used influenced the MCA, which was more significant at higher concentrations.

These results indicate that an increase in pH has a greater negative effect on the MCA of CC than that of CH and OP, and CH is the species whose MCA is the least affected by the increase in pH.

Other studies concluded that aqueous extracts obtained from flowers of *Cynara* species are more active at a slightly acidic pH [51]. Campos et al. (1990) [48] and Heimgartner et al. (1990) [27] previously demonstrated that the proteases, mainly endopeptidases, have a maximum clotting activity at pH values in the range of 5.1–5.7; these data are in accordance with our results. According to Sousa-Gallagher and Malcata (1996) [52] and Chen et al. (2003) [53], the maximum activity of an aqueous crude extract obtained from CC was seen at pH 5.9–6.0. However, Martínez and Esteban (1980) [30] reported that CH shows its highest clotting activity at pH 7.

To date, no data are available on OP; however, for a freeze-dried extract from *Onopordum tauricum*, a higher MCA was recorded at pH values ranging from 4.9 to 5.7 [25,26].

#### 3.3.4. Effect of Thistle Species and CaCl_2_

**Figure 4:**
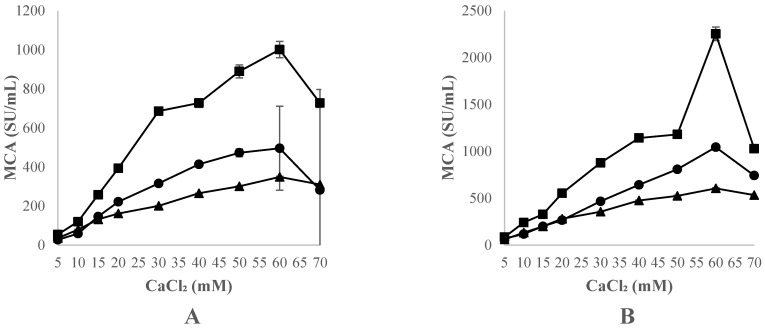
Effect of the concentration of CaCl_2_, expressed in mM, on the clotting activity (MCA), expressed in U/mL, of standard bovine skimmed milk at 32 °C and pH 6.2, using CC, (▲) CH (■) and OP (●), at the concentrations of 20 mg extract/mL (**A**) and 40 mg extract/mL (**B**). Data are mean of three independent experiments (*n* = 3). Error bars correspond to standard deviations.

The effect of adding CaCl_2_ at different concentrations (5, 10, 15, 20, 30, 40, 50, 60 and 70 mM) on milk at a temperature of 32 °C and a pH of 6.2 is shown in Figure 4.

For this purpose, three freeze-dried crude extracts (CC, CH, and OP) were assayed at concentrations of 20 and 40 mg/mL. As a general trend, MCA increased at higher CaCl_2_ concentrations for all thistle extracts assayed (*p* < 0.001). As far as the extract concentration is concerned, all the assayed species showed higher MCA values at 40 mg/mL than at 20 mg/mL.

All the species presented a maximum MCA value at 60 mM CaCl_2_ and at an extract concentration of 40 mg/mL. By comparing the three thistle species, the MCA of CH, at both extract concentrations, was significantly higher than that of CC and OP (*p* < 0.05) at all CaCl_2_ concentrations tested. The MCA of OP at 20 mg/mL was higher (*p* < 0.05) than that of CC at CaCl_2_ concentrations between 20–60 mM. At a 40 mg/mL extract concentration, the MCA of OP was significantly higher than the MCA of CC except at CaCl_2_ concentrations between 30 and 70 mM.

These results indicate that the addition of CaCl_2_ significantly improved the clotting activity of these freeze-dried crude extracts. Similar evidence emerges from other studies on aqueous extracts from CC and CH, showing that an increase in CaCl_2_ concentration leads to an improvement in clotting activity [30,44,54]. Nevertheless, based on the data herein collected, the MCA of the freeze-dried extract from CC was enhanced the least by an increase in CaCl_2_ concentration, compared to that of CH and OP; in contrast, the performance of these latter two species was most positively affected by the addition of high concentrations of CaCl_2_.

In the first phase of the coagulation process, once most of the Phenylalanine_105_–Methionine_106_ bonds have been cleaved, Ca^2+^ ions combine with para-kappa casein fractions to form firm clots. For this reason, the addition of CaCl_2_ to milk reduces the coagulation time and allows the aggregation of casein micelles [25,55,56,57].

### 3.4. Proteolytic Activity and MCA/PA Ratio

**Figure 5:**
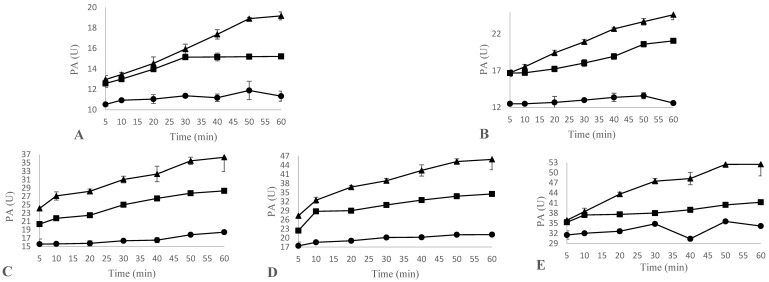
Effect of species CC (▲), CH (■), and OP (●) on the proteolytic activity (PA) of bovine casein at 32 °C and pH 6.2, expressed in U, at the concentrations of 5 mg extract/mL (**A**), 10 mg extract/mL (**B**), 20 mg extract/mL (**C**), 30 mg extract/mL (**D**) and 40 mg extract/mL (**E**). Data are the means of three independent experiments (n = 3). Error bars correspond to standard deviations. To the authors’ knowledge, no data are currently available on the performance of *Onopordum platylepis* for casein hydrolysis. Its enzymatic composition and the specificity of its component proteases are also unknown; nevertheless, the proteolytic activity of other species belonging to the genus *Onopordum*, such as *Onopordum acanthium* [47] and *Onopordum tauricum* [26], has previously been evaluated, showing a higher specificity for β- and α-caseins and lower specificity for κ-caseins. More specifically, the *Onopordum acanthium* enzymatic extract was shown to have a lower PA than chymosin and the *Cynara cardunculus* extract was.

The proteolytic activity (U) in bovine casein (32 °C and pH 6.2) at the reaction times (5, 10, 20, 30, 40, 50 and 60 min) for each of the extract concentrations assayed (5, 10, 20, 30 and 40 mg extract/mL) are shown in Figure 5. For the calculation of the MCA/PA ratio, the MCA values obtained at 32 °C, pH 6.2 and a concentration of 10 mM of CaCl_2_, and the PA values obtained at 60 min were considered (Table 2).

Comparison data referring to proteolytic activity between the different species, concentrations of extracts, and reaction times are given in the (Supporting Information Supplementary Tables S5 and S6).

The effects of the species on the proteolytic activity were compared at the same extract concentrations previously evaluated for the MCA. As shown in Figure 5, significant differences emerged by comparing species and the reaction time at a specific extract concentration (*p* < 0.001).

The CC and CH species reached a maximum PA value at the 40 mg/mL extract concentration and at 60 min of the reaction, respectively, these values being 52.49 U and 41.24 U. Regarding OP, the highest PA value (34.18 U) was recorded at an extract concentration of 40 mg/mL and at 50 min of hydrolysis.

As previously suggested, the increase in reaction time and extract concentration favourably affects the hydrolysis rate [31,33]. This is consistent with the results herein obtained.

It would be crucial to find the right extract concentration as an excess of proteolytic enzymes can increase secondary proteolysis related to bitter flavours, and an insufficient amount of these enzymes can affect the texture of the cheese by decreasing its consistency [58].

As far as the increase in hydrolysis rate is concerned, the extract obtained from CC showed the highest rate of hydrolysis at the different extract concentrations assayed, although in some cases, CH and CC extracts showed comparable activities. The slight difference that emerged in the proteolytic activity of CC and CH might be due to the fact that both species have in common the occurrence of an aspartic protease known as cardosin A; however, CC has a second protease called cardosin B, which is even more proteolytic [59].

As a general rule, a balanced degradation of caseins is necessary to develop favourable organoleptic characteristics in cheese. An excess of proteolytic activity during cheesemaking is associated with a low yield and an intense bitter taste due to the accumulation of small low-molecular-weight peptides and hydrophobic peptides responsible for the bitter taste [47,60].

Comparing the values obtained by the different species, the CC and CH species showed a better MCA/PA relation than the OP species did under the conditions tested in this study. Brutti et al. (2012) [47] found higher MCA and PA in the *Cynara cardunculus* extract than in *Onopordum acanthium;* however, the MCA/PA ratio of onopordosin was higher.

The MCA/PA ratio is an important measure related to higher cheese yield and quality. Therefore, the species with the highest MCA/PA ratio is the most suitable for use in cheesemaking. However, it would be advisable to explore the effect of various factors on the MCA/PA ratio, and the optimal conditions of extract concentration, temperature, pH and CaCl_2_ at which the performance of the extracts is increased should be taken into account.

## 4. Conclusions

The performance of the freeze-dried thistle extracts against variations in the factors involved in MCA and PA was as expected. The effect of the parameters (temperature, pH, and CaCl_2_ concentration) on the MCA of the extracts was similar at two extract concentrations (20 and 40 mg/mL). The results clearly showed the stability of these extracts at elevated temperatures, showing clotting activity up to a maximum temperature of 70 °C. An increase in pH adversely affects milk coagulation, but an increase in the extract concentration and the addition of CaCl_2_ improve this activity. Furthermore, proteolytic activity in bovine casein increased at higher extract concentrations and longer hydrolysis times.

To summarize, the milk clotting conditions to achieve maximum values for CH and OP are [extract] = 40 mg/mL, T = 70 °C, pH 5, and 60 mM CaCl_2_, and [extract] = 40 mg/mL, T = 70 °C, pH 5.5, and 60 mM CaCl_2_, respectively. The extract from CH showed similar behaviour to that obtained from CC in the hydrolysis of bovine casein, while OP was the species with the lowest caseinolytic capacity.

Given these points, CH and OP proved to be good milk coagulants, at least at the laboratory level, for cheese production. Nevertheless, further research on the use of these thistle-based coagulants in cheesemaking is ongoing to evaluate the changes they generate in proteolysis and the sensory characteristics of final cheeses.

## Figures and Tables

**Table 1:** Mineral composition (mg/100 g dry weight) of the freeze-dried extracts of *Cynara humilis* L. (CH) and *Onopordum platylepis* Murb. (OP).

Minerals	Species	
	CH	OP
Ca	346.1 ± 2.3 ^a^	330.7 ± 4.1 ^b^
P	638.9 ± 7.6 ^b^	778.1 ± 13.3 ^a^
Na	77.3 ± 3.4 ^a^	73.6 ± 1.5 ^a^
K	7577.7± 156.2 ^a^	5918.6 ± 86.4 ^b^
Mg	393.3 ± 0.4 ^a^	311.1 ± 1.4 ^b^
Zn	3.2 ± 0.1 ^a^	2.5 ± 0.1 ^b^
Mn	2.0 ± 0.0 ^b^	2.2 ± 0.1 ^a^

Data are mean ± standard deviation (n = 3). ^a,b^ different superscript letters in a row mean significant differences (HSD test, *p* < 0.05). CH, *Cynara humilis* L.; OP, *Onopordum platylepis* Murb.

**Table 2:** Milk clotting activity and proteolytic activity ratio (MCA/PA) of the species at different extract concentrations.

[Extract] (mg/mL)	MCA/PA ^1^		
	CC	CH	OP
5	3.2740	2.7359	1.8765
10	3.3861	3.2477	2.5854
20	4.3226	6.0250	3.4263
30	7.3651	7.0204	4.6564
40	7.7977	7.2768	3.5884

^1^ MCA expressed in SU/mL; PA expressed in U. [Extract], extract concentration; CC, *Cynara cardunculus* L.; CH, *Cynara humilis* L.; OP, *Onopordum platylepis* Murb.

## Data Availability

Data is contained within the article or supplementary material.

## Supplementary Materials

The following supporting information can be downloaded at: https://www.mdpi.com/article/10.3390/foods12122325/s1, Table S1: Milk clotting time (seconds) for milk coagulation of vegetable coagulants from Cynara cardunculus, Cynara humilis and Onopordum platylepis at different extract concentrations; Table S2: Effect of temperature (°C) on the MCT (seconds) of the vegetable coagulants from Cynara cardunculus, Cynara humilis, and Onopordum platylepis at different extract concentrations (mg/mL); Table S3: Effect of the pH on the MCT (seconds) of the vegetable coagulants from Cynara cardunculus, Cynara humilis, and Onopordum platylepis at different extract concentrations (mg/mL); Table S4: Effect of calcium chloride concentration (CaCl2) on the MCT (seconds) of the vegetable coagulants from Cynara cardunculus, Cynara humilis, and Onopordum platylepis at different extract concentrations (mg/mL); Table S5: Effect of species and reaction times (minutes) on the proteolytic activity (Uabs) of Cynara cardunculus, Cynara humilis, and Onopordum platylepis plant extracts at different extract concentrations (mg/mL); Table S6: Effect of species and extract concentrations (mg/mL) on the proteolytic activity (Uabs) of Cynara cardunculus, Cynara humilis, and Onopordum platylepis plant extracts at different reaction times (minutes).
