# Supplementary material for: Clotting and Proteolytic Activity of Freeze-Dried Crude Extracts Obtained from Wild Thistles Cynara humilis L. and Onopordum platylepis Murb
Source: Foods. 2023 Jun 9;12(12):2325. doi: 10.3390/foods12122325 (PMC10296898; doi:10.3390/foods12122325)
Supplement: Supplementary file 1 [file foods-12-02325-s001.zip › foods-2423229-supplementary.pdf]

Table S1. Milk clotting time (seconds) for milk coagulation of vegetable coagulants from Cynara *cardunculus*, Cynara *humilis* and Onopordum *platylepis* at different extract concentrations

[Extract] (mg/mL)	MCT (s) [1]		
	CC	CH	OP
5	382	577	1129
10	288	351	737
20	153	141	380
30	71	99	244
40	59	80	196

[1]Average values of three replicates.

[Extract] = extract concentration, MCT = milk clotting time

Table S2. Effect of temperature (ºC) on the MCT (seconds) of the vegetable coagulants from Cynara *cardunculus*, Cynara *humilis*, and Onopordum *platylepis* at different extract concentrations (mg/mL)

[Extract] (mg/mL)	T (°C)	MCT (s) [1]		
		CC	CH	OP
	20	2042	780	1404
	25	898	515	665
	30	421	302	387
	35	214	171	266
	40	137	106	150
**20**	45	80	60	105
	50	61	46	53
	60	37	28	35
	70	23	15	20
	80	4200	360	24
	85	5395	540	120
	20	1680	661	810
	25	599	357	379
	30	259	153	230
	35	130	95	157
	40	95	57	95
**40**	45	58	33	50
	50	40	25	38
	60	17	14	12
	70	10	10	6
	80	3905	179	3605
	85	4503	420	4306

[1]Average values of three replicates.
[Extract] = extract concentration, T = temperature, MCT = milk clotting time

Table S3. Effect of the pH on the MCT (seconds) of the vegetable coagulants from Cynara *cardunculus*, Cynara *humilis*, and Onopordum *platylepis* at different extract concentrations (mg/mL)

[Extract] (mg/mL)	pH	MCT (s) [1]		
		CC	CH	OP
20	5	10	147	271
	5.5	30	152	257
	6	65	157	294
	6.5	293	160	295
	7	1739	162	297
	7.5	3180	167	312
	8	7200	178	327
40	5	10	105	192
	5.5	25	106	172
	6	58	108	185
	6.5	102	108	190
	7	1195	108	195
	7.5	1801	114	195
	8	3000	119	215

[1]Average values of three replicates.
[Extract] = extract concentration, MCT = milk clotting time

Table S4. Effect of calcium chloride concentration ($CaCl_2$) on the MCT (seconds) of the vegetable coagulants from Cynara *cardunculus*, Cynara *humilis*, and Onopordum *platylepis* at different extract concentrations (mg/mL)

[Extract] (mg/mL)	[$CaCl_2$] (mM)	MCT (s) [1]		
		CC	CH	OP
20	5	658	440	869
	10	301	200	405
	15	182	93	164
	20	148	61	108
	30	119	35	76
	40	90	33	58
	50	80	27	105
	60	69	24	51
	70	77	33	48
40	5	390	279	339
	10	180	100	202
	15	120	73	120
	20	85	43	90
	30	67	27	51
	40	50	21	37
	50	46	20	30
	60	40	11	23
	70	45	23	32

[1]Average values of three replicates.

[Extract] = extract concentration, [$CaCl_2$] = calcium chloride concentration, MCT = milk clotting time

Table S5. Effect of species and reaction times (minutes) on the proteolytic activity (Uabs) of Cynara *cardunculus*, Cynara *humilis*, and Onopordum *platylepis* plant extracts at different extract concentrations (mg/mL).

[Extract]	Time	PA (Uabs 280 nm)[1]		
		CC	CH	OP
5	5	0.3239[hi]	0.3143[i]	0.2632[l]
	10	0.3360[gh]	0.3248[hi]	0.2732[kl]
	20	0.3631[ef]	0.3491[fg]	0.2760[kl]
	30	0.3983[c]	0.3784[de]	0.2841[jk]
	40	0.4342[b]	0.3789[d]	0.2793[k]
	50	0.4725[a]	0.3797[d]	0.2971[j]
	60	0.4793[a]	0.3803[d]	0.2832[jk]
10	5	0.4180[h]	0.4167[h]	0.3124[j]
	10	0.4379[fg]	0.4176[h]	0.3125[j]
	20	0.4852[e]	0.4309[gh]	0.3171[j]
	30	0.5227[d]	0.4508[f]	0.3248[ij]
	40	0.5669[c]	0.4733[e]	0.3342[i]
	50	0.5913[b]	0.5148[d]	0.3397[i]
	60	0.6150[a]	0.5264[d]	0.3148[j]
20	5	0.6030[fg]	0.5093[i]	0.3892[l]
	10	0.6776[cd]	0.5439[hi]	0.3907[l]
	20	0.7050[c]	0.5619[gh]	0.3941[l]
	30	0.7760[b]	0.6247[ef]	0.4092[kl]
	40	0.8087[b]	0.6628[de]	0.4129[kl]
	50	0.8867[a]	0.6940[cd]	0.4454[jk]
	60	0.9084[a]	0.7081[c]	0.4608[j]
30	5	0.6833[i]	0.5608[jk]	0.4352[n]
	10	0.8118[f]	0.7194[hi]	0.4638[mn]
	20	0.9200[d]	0.7250[hi]	0.4765[lm]
	30	0.9722[c]	0.7725[g]	0.5037[kl]

	40	1.0572^{b}	0.8128^{f}	0.5053^{kl}
	50	1.1300^{a}	0.8446^{ef}	0.5264^{jk}
	60	1.1480^{a}	0.8634^{ef}	0.5274^{jk}
40	5	0.8979^{gh}	0.8825^{h}	0.7881^{jk}
	10	0.9619^{f}	0.9366^{fg}	0.8010^{jk}
	20	1.0911^{c}	0.9415^{fg}	0.8159^{ij}
	30	1.1876^{b}	0.9509^{f}	0.8706^{h}
	40	1.2071^{b}	0.9751^{ef}	0.7600^{k}
	50	1.3108^{a}	1.0115^{de}	0.8889^{h}
	60	1.3122^{a}	1.0311^{d}	0.8546^{hi}

[1]Different superscript letters mean significant differences (LSD test, $p < 0.05$). Data are mean of three experiments.
[Extract] = extract concentration, PA = proteolytic activity

Table S6. Effect of species and extract concentrations (mg/mL) on the proteolytic activity (Uabs) of Cynara *cardunculus*, Cynara *humilis*, and Onopordum *platylepis* plant extracts at different reaction times (minutes).

**Time**	**[Extract]**	**PA (Uabs 280 nm)[1]**		
		**CC**	**CH**	**OP**
5	5	0.3239^{i}	0.3143^{i}	0.2632^{j}
	10	0.4180^{gh}	0.4167^{gh}	0.3124^{i}
	20	0.6030^{d}	0.5093^{f}	0.3892^{h}
	30	0.6833^{c}	0.5608^{e}	0.4352^{g}
	40	0.8979^{a}	0.8825^{a}	0.7881^{b}
10	5	0.3360^{kl}	0.3248^{kl}	0.2732^{m}
	10	0.4379^{h}	0.4176^{i}	0.3125^{l}
	20	0.6776^{e}	0.5439^{f}	0.3907^{j}
	30	0.8118^{c}	0.7194^{d}	0.4638^{g}
	40	0.9619^{a}	0.9366^{b}	0.8010^{c}
20	5	0.3631^{i}	0.3491^{i}	0.2760^{k}
	10	0.4852^{f}	0.4309^{g}	0.3171^{j}
	20	0.7050^{d}	0.5619^{e}	0.3941^{h}
	30	0.9200^{b}	0.7250^{d}	0.4765^{f}
	40^{a}	1.0911	0.9415^{b}	0.8159^{c}
30	5	0.3983^{hi}	0.3784^{i}	0.2841^{k}
	10	0.5227^{f}	0.4508^{g}	0.3248^{j}
	20	0.7760^{d}	0.6247^{e}	0.4092^{hi}
	30	0.9722^{b}	0.7725^{d}	0.5037^{f}
	40	1.1876^{a}	0.9509^{b}	0.8706^{c}
40	5	0.4342^{ij}	0.3789^{k}	0.2793^{m}
	10	0.5669^{g}	0.4733^{hi}	0.3342^{l}
	20	0.8087^{d}	0.6628^{f}	0.4129^{jk}
	30	1.0572^{b}	0.8128^{d}	0.5053^{h}
	40	1.2071^{a}	0.9751^{c}	0.7600^{e}

50	5	0.4725[i]	0.3797[k]	0.2971[m]
	10	0.5913[g]	0.5148[h]	0.3397[l]
	20	0.8867[d]	0.6940[f]	0.4454[j]
	30	1.1300[b]	0.8446[e]	0.5264[h]
	40	1.3108[a]	1.0115[c]	0.8889[d]
60	5	0.4793[hi]	0.3803[j]	0.2832[k]
	10	0.6150[g]	0.5264[h]	0.3148[k]
	20	0.9084[de]	0.7081[f]	0.4608[i]
	30	1.1480[b]	0.8634[de]	0.5274[h]
	40	1.3122[a]	1.0311[c]	0.8546[e]

[1]Different superscript letters mean significant differences (LSD test, $p < 0.05$). Data are mean of three experiments.
[Extract] = extract concentration, PA = proteolytic activity
